# Single Cell Transcriptomic Atlas Reveals Cellular Heterogeneity and Molecular Mechanisms in Pediatric Airway Hyperresponsiveness Epithelium

**DOI:** 10.1155/ijog/3815412

**Published:** 2026-06-09

**Authors:** Weiming Yang, Shunfeng Mao, Liuyang Zhou

**Affiliations:** ^1^ Pediatric department, Affiliated Hospital of Jiaxing University, Jiaxing, China

**Keywords:** airway mucosa, cellular heterogeneity, macrophage subpopulations, network pharmacology, pediatric airway hyperresponsiveness, single-cell rna sequencing, xiao qing long tang

## Abstract

**Background:**

Airway hyperresponsiveness (AHR) is a clinically important feature of chronic pediatric inflammatory airway disease, but its cellular architecture remains incompletely resolved. Because bulk transcriptomic approaches can mask signals from rare or state‐specific cell populations, single‐cell RNA sequencing (scRNA‐seq) offers a useful framework for examining epithelial and immune cell diversity in hyperresponsive airways.

**Methods:**

Publicly available scRNA‐seq data (GEO: GSM8722221) from bronchial brushing specimens of pediatric AHR patients (10x Genomics Chromium platform) were processed with Cell Ranger, quality‐filtered, and analyzed using PCA (15 PCs), UMAP/tSNE embedding, and Leiden clustering (resolution = 0.8). Cell types were annotated by canonical markers and validated via Seurat label transfer. Pseudotime trajectories were inferred with Monocle (DDRTree) and validated by scVelo, Slingshot, and PAGA. Macrophage subpopulations were resolved at resolution = 1.2 and profiled by hdWGCNA. GO and KEGG enrichment used clusterProfiler (Benjamini‐Hochberg; adjusted p <0.05). Network pharmacology integrated TCM BTMAN 2.0, Swiss Target Prediction, SEA, and PPI topology to identify targets and herbal compounds from Xiao Qing Long Tang.

**Results:**

Quality control supported the analytical suitability of the dataset (mitochondrial fraction vs. nCount_RNA r = ‐0.31; inter‐dataset r = 0.96). Actb, Gapdh, Tuba1b, Hmga2, Vim, Col1a1, Zeb1, Snai2, Twist1, and Mmp9 were among the top variable genes, and the first 15 PCs were retained after JackStraw testing. UMAP and tSNE resolved eight airway mucosal cell populations with concordant separation. Cell‐type‐specific expression of TSLP, IL33, CCL26, S100A8, POSTN, CLCA1, FCGR3B, TNFRSF8, FOXJ1, and SCGB1A1 was consistent with known inflammatory, remodeling, and mucociliary programs. High‐resolution macrophage analysis identified 23 putative subpopulations, which were interpreted cautiously as computationally defined states. Enrichment analysis highlighted cytoplasmic translation, ribosome biogenesis, oxidative phosphorylation, and related mitochondrial signatures. Network pharmacology identified VEGFA, PTGS2, and ESR1 among prioritized targets, yielding 83 shared candidates between disease‐associated and compound‐associated target sets.

**Conclusions:**

This study provides a pediatric AHR single‐cell atlas and an integrated, hypothesis‐generating view of epithelial‐immune heterogeneity, macrophage state diversity, and candidate therapeutic networks. The findings should be interpreted as computational evidence that requires validation in healthy pediatric controls, independent cohorts, and functional experiments.

## 1. Introduction

Airway hyperresponsiveness (AHR) represents one of the most prevalent chronic respiratory disorders in children, affecting an estimated 300 million individuals worldwide, with incidence rising steadily across diverse geographic and socioeconomic settings [1, 2]. Pediatric AHR is clinically defined by the triad of airway inflammation, bronchial hyperreactivity, and progressive structural remodeling, manifesting as recurrent episodes of dyspnea, wheezing, chest tightness, and persistent cough [3]. Despite decades of research establishing foundational insights into AHR pathophysiology, the inherent cellular heterogeneity of hyperresponsive airways and the intricate interplay among genetic susceptibility loci, environmental insults, and dysregulated immune circuits continue to impede the translation of mechanistic knowledge into effective, personalized therapies.

The respiratory mucosa represents the primary interface between the host immune system and the inhaled environment, fulfilling indispensable roles in physical barrier maintenance, mucociliary clearance, and innate and adaptive immune modulation [4, 5]. In AHR patients, this mucosal layer undergoes profound pathological transformation encompassing epithelial barrier disruption, pathological mucus hypersecretion, impaired ciliary beat frequency, and dysregulated epithelial repair mechanisms [6]. These molecular and cellular alterations are mechanistically central to the cardinal clinical features of AHR and are likely to involve spatially and temporally coordinated changes across multiple interdependent cell types whose precise contributions remain poorly resolved.

Conventional bulk RNA‐sequencing has provided foundational transcriptional maps of AHR‐associated gene expression. However, this approach fundamentally obscures disease‐relevant signals by averaging across heterogeneous cell populations, introduces systematic biases through RNA extraction protocols, and cannot capture the microenvironmental context critical to cell‐cell communication [7]. Single‐cell RNA sequencing (scRNA‐seq) has transformed our capacity to interrogate disease processes with single‐cell resolution, enabling identification of rare cell populations, precise characterization of cell state transitions, and inference of developmental trajectories from snapshot transcriptional data [8, 9]. In the context of airway disease, scRNA‐seq has moved beyond the constraints of bulk approaches to resolve epithelial subtype‐specific transcriptional programs, enumerate functionally relevant rare populations such as ionocytes and deuterosomal cells, and uncover intercellular signaling axes that are masked by bulk averaging.

Emerging single‐cell investigations of asthma and related airway diseases have begun to reveal striking cellular diversity, identifying distinct epithelial subpopulations with disease‐specific molecular signatures and specialized functional roles. Nonetheless, comprehensive single‐cell atlases that map the full cellular ecosystem of pediatric AHR airway mucosa with integrated functional annotation remain notably lacking. The molecular determinants of disease‐specific cellular heterogeneity in this age‐specific context have yet to be systematically characterized.

The present study addresses this gap through detailed scRNA‐seq profiling of pediatric AHR airway mucosal tissues, with the following specific aims: (1) to construct a comprehensive cellular atlas of hyperresponsive airway mucosa; (2) to define cell‐type‐specific molecular signatures and marker genes; (3) to elucidate epithelial differentiation trajectories and lineage relationships; (4) to characterize macrophage subpopulation diversity and functional specialization at high resolution; (5) to identify disease‐associated transcriptional networks and upstream regulatory mechanisms; and (6) to integrate network pharmacology for therapeutic target prioritization.

## 2. shared nearest‐neighbor (SNN) graph was then bMethods

### 2.1. Study Design and Data Sources

Single‐cell RNA sequencing data from pediatric AHR airway mucosal tissue were obtained from the Gene Expression Omnibus (GEO) public repository (accession: GSM8722221). The dataset contains transcriptomic profiles of bronchial brushing specimens from pediatric AHR patients generated using the 10x Genomics Chromium platform. Bronchial brushing mainly samples the airway surface epithelium but can also recover immune cells present in the mucosa and airway lumen, particularly in inflamed samples. According to the original dataset description, CD45 depletion was not performed before library construction, allowing epithelial and immune compartments to be profiled together. Full cohort information, including sample size, age and sex distribution, diagnostic criteria, comorbidities, medication status, and specimen collection procedures, is provided in the original dataset documentation and Supporting Information Table S1 [10–12].

Raw sequencing reads were processed using Cell Ranger, which performed reference genome alignment, gene‐level UMI counting, and cellular barcode demultiplexing. Per‐cell quality metrics—number of detected genes, total RNA count (nCount_RNA), and mitochondrial gene fraction—were computed for every barcode. Bivariate scatter plots of nFeature_RNA versus nCount_RNA and of mitochondrial fraction versus nCount_RNA were inspected to assess data fidelity and inter‐sample consistency, and threshold‐based filters were applied to exclude low‐quality barcodes and putative doublets before downstream analysis.

### 2.2. Low‐Dimensional Representation of Transcriptomic Space Reveals Discrete and Reproducible Cell Clusters

PCA was performed on the scaled highly variable gene matrix. Fifty principal components were first calculated, and the number used for downstream analysis was selected using the elbow plot and the Kaiser criterion (eigenvalue >1). The first 15 PCs were retained, with JackStraw permutation testing supporting their statistical relevance (all p <0.05). A uilt in this 15‐dimensional PC space and used for both clustering and UMAP visualization. UMAP embedding (n_neighbors = 30, min_dist = 0.3) projected the SNN graph into two dimensions, while tSNE was performed independently to assess whether cluster separation was reproducible across embedding methods. Leiden graph‐based clustering (resolution = 0.8) partitioned the neighborhood graph into discrete communities, which were then used for cell‐type annotation and functional analysis.

### 2.3. Cell Type Identification and Annotation

Each Leiden cluster was annotated by overlaying expression of canonical lineage marker genes. Cell identity assignments were validated through three orthogonal approaches: (1) gene module scoring using published airway epithelial signatures; (2) reference‐based label transfer against established airway atlases implemented in Seurat; and (3) differential expression analysis to quantify marker specificity and minimize cross‐cluster ambiguity. This multi‐step procedure resolved seven distinct airway mucosal populations—eosinophils, basal cells, ciliated cells, goblet cells, club cells, ionocytes, and deuterosomal cells—each displaying a unique transcriptional signature consistent with its known biological identity. The relative abundance of each population was quantified across the full cellular atlas. Goblet cells and eosinophils, populations directly implicated in mucus hypersecretion and eosinophilic inflammation, were of primary pathological interest. Basal cells, which serve as the multipotent progenitor reservoir of the airway epithelium, constituted a substantial fraction of the atlas, consistent with their central role in epithelial regeneration and remodeling. The concurrent detection of ciliated and goblet cells at altered proportions reflects the mucociliary dysfunction characteristic of AHR airways.

### 2.4. Cell Fate Trajectories and Associated Transcriptional Dynamics Inferred from Single‐Cell Pseudotime Ordering

Pseudotime trajectories were reconstructed using Monocle with the DDRTree dimensionality reduction method, anchoring basal cells as the root state on the basis of their established multipotent progenitor identity. Trajectory directionality was independently validated by RNA velocity analysis with scVelo, which models transcriptional dynamics from the ratio of spliced to unspliced mRNA across cells. Consistency of the inferred developmental ordering was further cross‐checked with alternative algorithms, Slingshot and PAGA, to confirm robustness across computational frameworks. Pseudotime scores were assigned to each cell to quantify its relative position along the inferred differentiation continuum. It should be noted that pseudotime represents a computationally inferred transcriptional ordering and does not constitute experimentally verified lineage progression. Trajectory analysis revealed a continuous differentiation axis from multipotent basal progenitors toward specialized epithelial subtypes, with distinct transcriptional progression patterns observed under hyperresponsive pathological conditions.

Dynamic gene expression changes along the pseudotime axis were examined to identify modules with significant co‐variation during differentiation. Hierarchical clustering of co‐expressed genes delineated distinct expression modules, each representing coherent biological programs or regulatory circuits active at specific stages of epithelial lineage commitment. These gene modules illuminate the molecular events governing cell fate transitions and provide mechanistic insight into how chronic hyperresponsive inflammation perturbs normal epithelial differentiation programs.

### 2.5. In‐depth Analysis of Macrophage Subpopulations

Macrophages were isolated in silico and reclustered using a higher Leiden resolution (resolution = 1.2), producing 23 computationally defined subpopulations (M1‐M23). Because high‐resolution clustering can sometimes divide continuous or transitional cell states into smaller algorithmic groups, cluster interpretation was supported by four complementary checks: (1) hierarchical clustering into broader functional supergroups, including pro‐inflammatory, anti‐inflammatory, and tissue‐remodeling patterns; (2) subsampling‐based stability analysis; (3) silhouette score evaluation; and (4) comparison with published lung macrophage atlases where applicable. kME bar plots and violin plots of hub genes were then used to describe, rather than overstate, the molecular features of each subpopulation.

Functional polarization state of each subpopulation was characterized using classical M1 and M2 marker genes, enabling assignment of classically activated, alternatively activated, or intermediate phenotypes. These annotations were contextualized against current macrophage biology literature, including recently described tissue‐specific and context‐dependent activation states that extend beyond the canonical M1/M2 framework. The potential contributions of individual subpopulations to inflammatory amplification, tissue repair, fibrogenesis, and immune regulation were assessed, providing a functional heterogeneity map to guide the design of subpopulation‐targeted immunomodulatory strategies.

### 2.6. Network Pharmacology Analysis

Disease‐associated target genes were compiled from public databases using the core pathogenic genes and key cell populations identified by scRNA‐seq as a starting point. Compound‐target associations for Xiao Qing Long Tang were retrieved from TCM BTMAN 2.0 and further predicted using Swiss Target Prediction and SEA. The overlap between disease targets and compound targets yielded 83 shared therapeutic candidates. A protein‐protein interaction (PPI) network was constructed for these overlapping targets, and topological measures, including degree, betweenness, and closeness centrality, were used to prioritize VEGFA, PTGS2, ESR1, AKT1, TP53, TNF, IL6, STAT3, EGFR, and PIK3CA. Herb‐level target coverage, herb‐compound‐target networks, hierarchical clustering of herb‐target interaction profiles, and UpSet intersection analysis were used to explore potential multi‐component and multi‐target relationships. These analyses were treated as hypothesis‐generating because no molecular docking, perturbation assay, or animal validation was performed in the present study.

### 2.7. Functional Enrichment Analysis and Pathway Resolution

GO enrichment analysis was performed on differentially expressed gene sets across all identified cell clusters, covering three annotation domains: Biological Process, Cellular Component, and Molecular Function. The hypergeometric test assessed enrichment significance, with Benjamini‐Hochberg correction applied across all tested terms (adjusted p <0.05). BP analysis prioritized cytoplasmic translation, ribosome assembly, ATP metabolic process, ribonucleoprotein complex biogenesis, oxidative phosphorylation, aerobic respiration, and ribosome biogenesis. CC analysis highlighted cytosolic ribosome, ribosomal subunit, focal adhesion, and cell‐substrate junction. MF analysis identified structural constituent of ribosome, cadherin binding, rRNA binding, and NADH dehydrogenase activities as significant functional categories.

KEGG pathway enrichment was then performed to map differentially expressed genes onto canonical signaling pathways, identifying Ribosome, Coronavirus disease‐COVID‐19, Parkinson disease, Oxidative phosphorylation, Huntington disease, Prion disease, Chemical carcinogenesis‐ROS, Diabetic cardiomyopathy, Amyotrophic lateral sclerosis, and Alzheimer disease as the most significantly enriched pathways [13–15]. A circular inter‐functional module association network was constructed in which nodes represent genes and arcs encode multi‐pathway membership, with arc thickness reflecting log2 fold‐change magnitude. KEGG dot plots summarized enrichment statistics (gene count and adjusted *p*‐value) for all significantly enriched pathways.

### 2.8. Batch Effect Correction and Integration

Data from pediatric AHR patients were integrated using the Harmony algorithm to remove inter‐sample batch effects while preserving biologically meaningful transcriptional variation. Correction efficacy was quantified by three metrics: (1) k‐nearest neighbor batch effect test (kBET) score; (2) local inverse Simpson’s index (LISI); and (3) silhouette coefficient. Pre‐ and post‐integration UMAP embeddings were compared to confirm adequate cell mixing across samples without collapsing biologically distinct populations. Retention of biological signal was verified by confirming that canonical cell‐type marker genes maintained consistent cluster‐level expression patterns after integration.

## 3. Statistical Analysis

All analyses were conducted in R (version ≥4.0). Single‐cell data processing and cell clustering used the Seurat package; UMAP and tSNE embeddings provided complementary two‐dimensional visualizations [16, 17]. Leiden graph‐based clustering (resolution = 0.8 for whole‐dataset; resolution = 1.2 for macrophage sub‐clustering; UMAP n_neighbors = 30, min_dist = 0.3) generated discrete cell communities. Pseudotime trajectory analysis used Monocle with the DDRTree method and default pipeline settings. Functional enrichment analysis was performed with the clusterProfiler package. Differentially expressed genes between clusters were identified by Wilcoxon rank‐sum test and defined as|log2 fold‐change| ≥1 with Benjamini‐Hochberg‐adjusted *p*‐value <0.05; the same multiple‐testing correction was applied to all GO and KEGG enrichment analyses. Results were visualized as scatter plots, violin plots, dot plots, heatmaps, volcano plots, and network diagrams to optimize interpretability and biological clarity.

## 4. Results

### 4.1. From Raw Transcriptomes to Interpretable Cell Landscapes: Preprocessing and Dimensionality Reduction of Pediatric AHR scRNA‐seq Data

**Figure 1:**
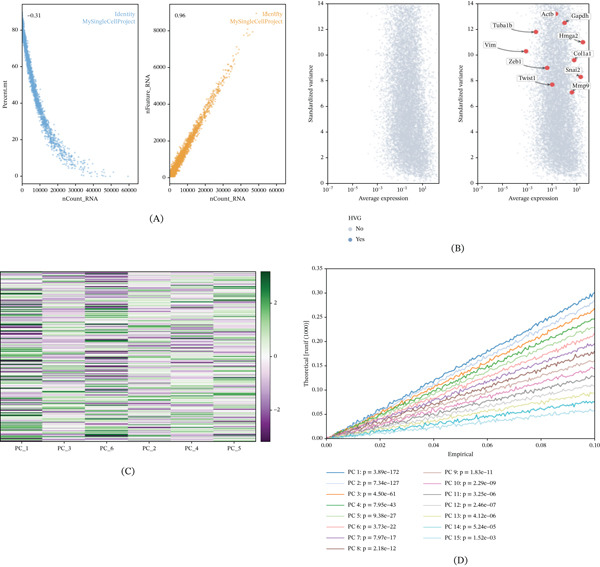
Establishment of a high‐quality single‐cell transcriptomic framework for interrogating pediatric airway hyperresponsiveness through quality control and dimensionality reduction. (A) Scatter plots showing mitochondrial gene percentage versus total RNA counts (nCount_RNA) per cell, with a correlation of −0.31, and detected gene number (nFeature_RNA) versus nCount_RNA, with a correlation of 0.96, confirming appropriate single‐cell quality characteristics and effective batch‐effect management. (B) Highly variable gene identification plots before and after annotation, highlighting representative variable genes including Actb, Gapdh, Tuba1b, Hmga2, Vim, Col1a1, Zeb1, Snai2, Twist1, and Mmp9. (C) Hierarchical clustering heat map of gene expression across the first six principal components (PC_1 through PC_6), demonstrating distinct transcriptional patterns stratifying cell populations. (D) JackStraw permutation test plot showing statistical significance of the first 15 principal components and supporting dimensionality reduction for downstream clustering and trajectory analysis.

Quality control of the scRNA‐seq dataset supported its suitability for downstream analysis. Figure 1A–1D summarizes the distribution of RNA count and feature‐level metrics. The mitochondrial fraction showed a modest inverse relationship with total RNA counts (r = ‐0.31), while the comparison of detected genes and total counts showed strong internal consistency (r = 0.96). Together, these patterns suggested that low‐quality barcodes and obvious technical outliers had been adequately controlled before dimensionality reduction and clustering.

### 4.2. Single‐cell Atlas Development and Cellular Classification of Pediatric Airway Hyperresponsiveness Mucosal Tissues

**Figure 2:**
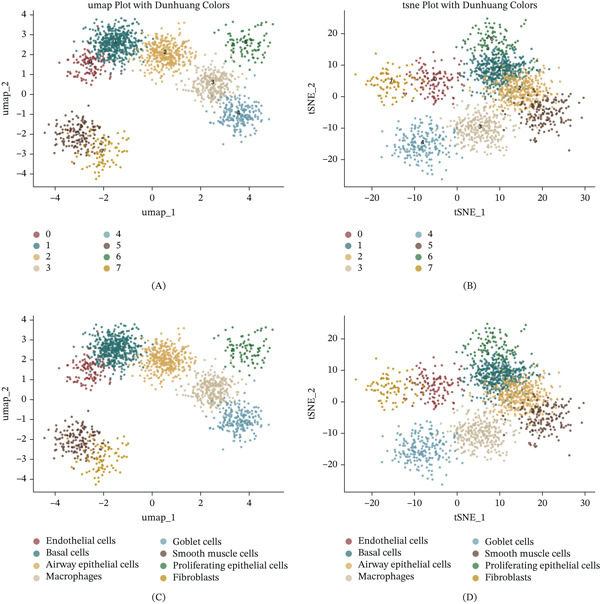
UMAP and tSNE dimensionality reduction analysis and cell‐type identification in pediatric airway hyperresponsiveness mucosal cells. (A) UMAP plot with numerical cluster labels (0–7) using Dunhuang‐inspired color palette, showing spatial segregation of transcriptionally distinct cell populations. (B) tSNE plot with the same numerical cluster labels demonstrating consistency of cell population separation across dimensionality reduction algorithms. (C) UMAP plot annotated with cell‐type identities based on canonical marker gene expression. (D) tSNE plot with identical cell‐type annotations confirming the robustness of cellular identification independent of the dimensionality reduction method employed.

UMAP dimensionality reduction was used to visualize transcriptional similarity among airway mucosal cells from pediatric AHR samples. As shown in Figures 2A and 2B, cells separated into discrete regions, with each color representing a transcriptionally related cluster. The broadly similar separation observed by UMAP and tSNE indicates that the major cell groups were not dependent on a single visualization method.

Marker‐based annotation (Figures 2C and 2D) identified the major airway mucosal populations represented in the dataset, including epithelial, endothelial, basal, goblet, ciliated, and immune‐enriched compartments. Each annotated population occupied a coherent region in UMAP and tSNE space, supporting the reliability of the cell‐type assignment. The presence of goblet‐cell and macrophage‐enriched compartments is consistent with mucus dysregulation and innate immune activation in AHR, although disease specificity cannot be established without matched healthy pediatric controls.

### 4.3. Spatially Resolved Gene Expression Signatures Delineating Cellular Identity and Inflammatory Circuitry in Pediatric AHR Mucosal Tissues

**Figure 3:**
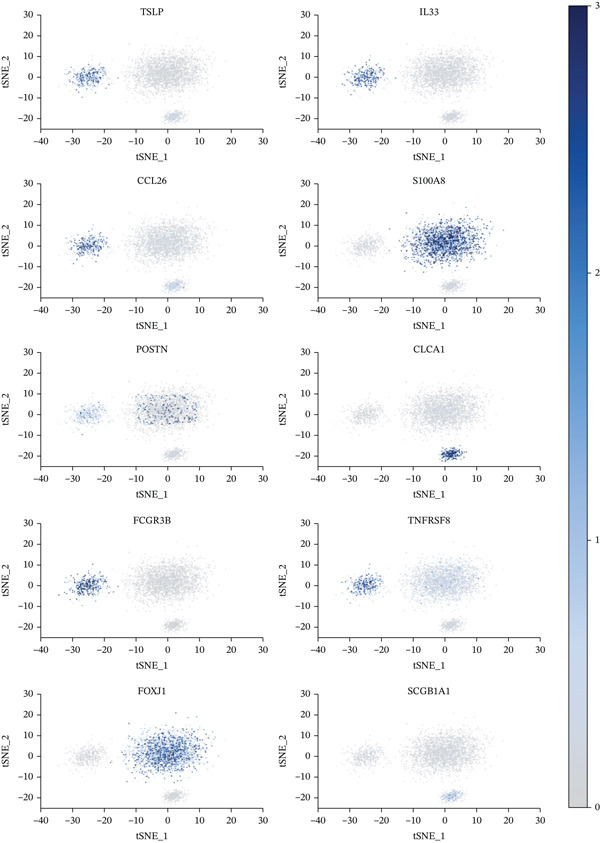
Single‐cell expression landscape of key genes in pediatric airway mucosal cells with airway hyperresponsiveness. Ten panels display individual gene expression distributions in t‐SNE space. Blue‐to‐dark‐navy dots indicate elevated expression on a scale of 0–3, whereas gray dots indicate low or absent expression. The genes shown are TSLP, an epithelial alarmin driving type 2 inflammation; IL33, an interleukin‐33 alarmin cytokine activating innate lymphoid cells; CCL26, an eotaxin‐3 eosinophil recruitment chemokine enriched in the immune cluster; S100A8, a calcium‐binding alarmin broadly expressed as an innate immune marker; POSTN, periostin, a subepithelial fibrosis and remodeling marker; CLCA1, a chloride channel accessory protein associated with secretory epithelium; FCGR3B, Fc gamma receptor IIIb, a neutrophil surface marker enriched in the immune cluster; TNFRSF8, CD30, an immune cell activation receptor; FOXJ1, a master transcriptional regulator of ciliated cell differentiation; and SCGB1A1, secretoglobin 1A1, a club cell secretory protein marker.

When projected onto the tSNE embedding, the ten selected genes showed structured but heterogeneous expression across pediatric AHR airway mucosal cells (Figure 3). TSLP and IL33 were enriched in a restricted epithelial‐associated region, consistent with their known roles as upstream alarmins in type 2 airway inflammation. CCL26 expression in a related region suggested a possible link to eosinophil recruitment, while S100A8 and FCGR3B marked immune‐enriched areas compatible with myeloid and neutrophil‐associated activity. POSTN showed a broader stromal‐like distribution, consistent with remodeling‐related extracellular matrix signaling, whereas CLCA1 was concentrated in a compact secretory epithelial cluster. FOXJ1 and SCGB1A1 further supported ciliated and club‐cell annotation, respectively. Overall, these expression patterns mainly reinforce established inflammatory and epithelial‐remodeling programs, while providing a cell‐resolved map of where those programs are represented within the pediatric AHR mucosal dataset.

### 4.4. Lineage Commitment and Transcriptional Specialization in Pediatric Airway Hyperresponsiveness Mucosal Tissues

**Figure 4:**
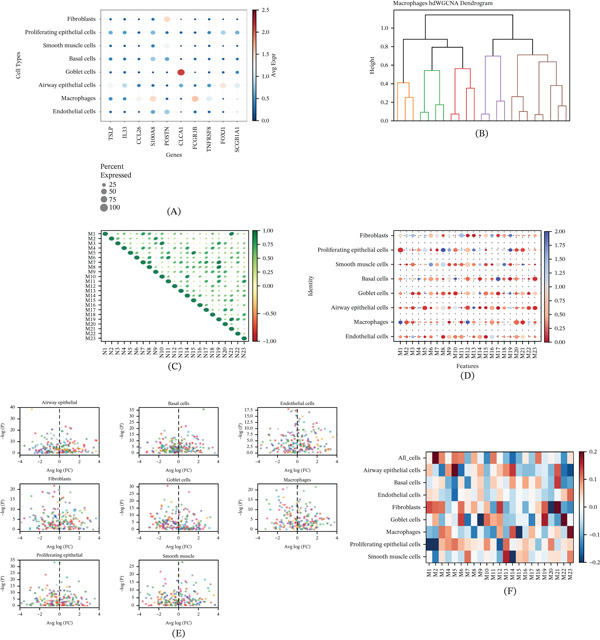
Integrated transcriptomic profiling and macrophage subpopulation analysis in pediatric airway hyperresponsiveness mucosal cells. (A) Dot plot of cell‐type‐specific marker gene expression for ten key AHR‐associated genes: TSLP, IL33, CCL26, S100A8, POSTN, CLCA1, FCGR3B, TNFRSF8, FOXJ1, and SCGB1A1. Dot size = proportion of expressing cells; color intensity = average expression level (blue = high, red = low). (B) hdWGCNA hierarchical dendrogram of macrophage gene co‐expression modules, clustering genes by co‐expression similarity with colored bars indicating distinct module assignments across the macrophage transcriptome. (C) Macrophage subpopulation correlation matrix (M1–M23). Ellipse orientation and color intensity (green = positive; purple = negative correlation) indicate transcriptional similarity between subpopulations; asterisks denote statistically significant correlations (p <0.05). (D) Dot plot of macrophage subpopulation (M1–M23) marker expression across major cell types, quantifying cell‐type‐enriched expression patterns of macrophage module genes. (E) Volcano plots of differentially expressed genes across eight cell types. *x*‐axis = average log2 fold‐change; *y*‐axis = −log10 (adjusted *p*‐value). Colored dots represent macrophage module membership, revealing cell‐type‐specific transcriptional programs. (F) Correlation heat map showing associations between macrophage subpopulations (M1–M23) and quality metrics (nFeature_RNA, nCount_RNA) across cell types (color scale: −0.2 to +0.2).

Differential gene expression analysis (Figure 4A) supported the cell‐type annotation framework by identifying subtype‐enriched marker genes. The dot plot simultaneously shows the proportion of cells expressing each marker and the average expression level within each cluster. Microvascular endothelial cells, ciliated cells, basal cells, and goblet cells displayed distinct marker profiles, providing molecular support for the annotated cellular identities.

Pseudotime analysis using Monocle (Figure 4B) placed basal cells near the inferred root because of their recognized progenitor role in the airway epithelium. The resulting trajectory suggested gradual transcriptional transitions from basal‐like states toward more differentiated epithelial populations. However, because this analysis is based on cross‐sectional transcriptomic data, the trajectory should be viewed as an inferred ordering of cell states rather than direct evidence of lineage progression.

Heatmap analyses (Figures 4C and 4D) added temporal and intercellular context to these inferred transitions. In Figure 4C, genes were grouped into modules along the pseudotime axis, highlighting coordinated expression changes that may accompany epithelial specialization. Figure 4D summarizes pairwise expression relationships between cell types, helping to identify co‐regulated programs that may operate across, rather than within, individual lineages.

Figure 4E extends the inferred trajectory into two‐dimensional space, with cells colored by differentiation state and trajectory lines indicating possible routes of transcriptional transition. Figure 4F then links the gene modules to broader biological processes, including cell fate specification, inflammatory signaling, and airway remodeling. These analyses provide a useful framework for hypothesis generation, but causal effects on epithelial repair or disease persistence will require functional validation.

### 4.5. Deconstructing Macrophage Diversity: Subpopulation Architecture and Lineage‐Specific Functions in Pediatric Airway Hyperresponsiveness

**Figure 5:**
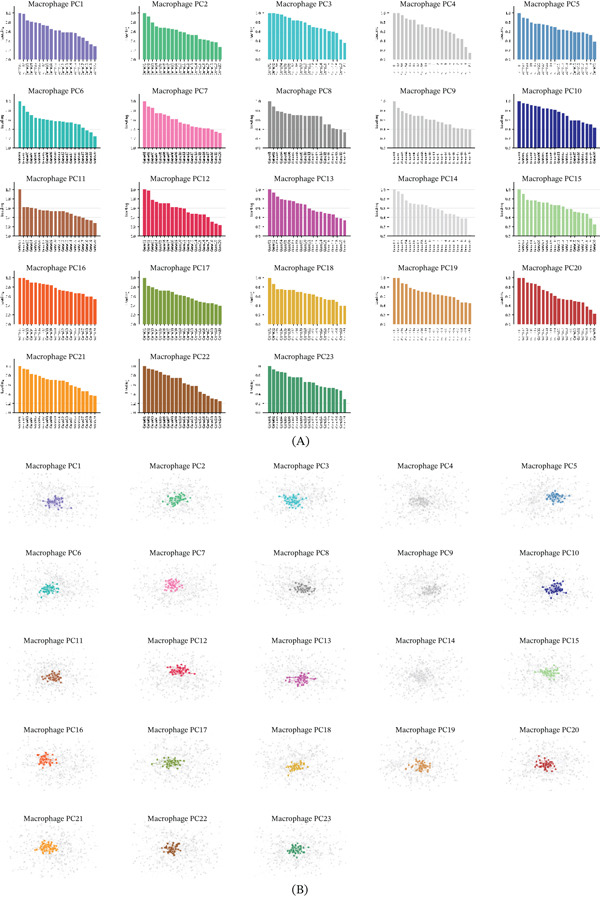
Transcriptional module profiles and spatial distribution of 23 macrophage subpopulations in pediatric airway hyperresponsiveness. (A) kME (module eigengene‐based connectivity) bar plots for each of the 23 macrophage subpopulations (M1–M23). Each panel shows top 15 hub genes (drawn from AHR‐relevant transcription factors, signaling molecules, and immune regulators including VEGFA, PTGS2, ESR1, AKT1, TP53, TNF, IL6, STAT3, EGFR, PIK3CA, and related genes) with their kME score distribution; colors distinguish subpopulation identities. Subpopulations are functionally classified into pro‐inflammatory (M1, M2, M6, M8, M10, M13, M16), anti‐inflammatory (M3, M5, M7, M9, M15, M19, M21), and tissue‐remodeling (M4, M11, M12, M14, M17, M18, M20, M22, M23) categories. (B) UMAP feature plots for each of the 23 macrophage subpopulations. Each panel shows spatial density and module score intensity (+ high, − low) within the total cellular UMAP landscape, with cohesive clusters indicating functionally specialized subpopulations.

High‐resolution single‐cell analysis identified 23 macrophage‐associated subpopulations (M1‐M23) in pediatric AHR airway mucosal tissue. These groups were organized into broader functional categories by hierarchical clustering, including pro‐inflammatory, anti‐inflammatory, and tissue‐remodeling patterns. Figure 5A shows the characteristic gene expression profiles of these subpopulations, with violin plots illustrating variation in inflammation‐related, remodeling‐related, and metabolism‐related genes. Although this classification captures macrophage transcriptional diversity, some small or partially overlapping clusters may reflect transitional states or clustering resolution effects rather than stable biological subtypes.

UMAP visualization (Figure 5B) shows the distribution of macrophage‐associated subpopulations within the broader cellular landscape. Several subpopulations occupied compact regions, suggesting shared transcriptional programs, whereas others appeared more dispersed and may represent intermediate or context‐dependent activation states. These spatial patterns provide clues about macrophage functional diversity in the AHR microenvironment, but the biological independence of each subpopulation should be confirmed through external datasets and experimental assays.

### 4.6. At the Intersection of Inflammation and Airway Remodeling: Pathway Enrichment and Network‐Level Functional Analysis of Pediatric AHR Cells

**Figure 6:**
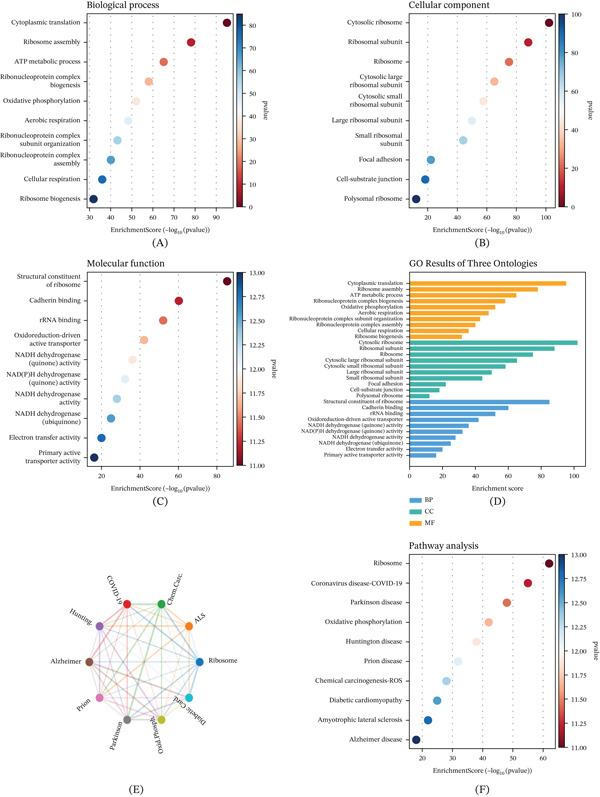
Mapping dysregulated biological programs and their pathway‐level interactions across cell types in pediatric airway hyperresponsiveness. (A) GO Biological Process enrichment dot plot. Top terms: cytoplasmic translation, ribosome assembly, ATP metabolic process, ribonucleoprotein complex biogenesis, oxidative phosphorylation, aerobic respiration, ribonucleoprotein complex subunit organization, and ribosome biogenesis. Dot size = gene count; color = *p*‐value. (B) GO Cellular Component enrichment. Top terms: cytosolic ribosome, ribosomal subunit, ribosome, focal adhesion, and cell‐substrate junction. (C) GO Molecular Function enrichment. Top terms: structural constituent of ribosome, cadherin binding, rRNA binding, and NADH dehydrogenase activities. (D) Integrated horizontal bar chart combining all three GO ontology categories (BP = orange, CC = teal, MF = blue), displaying comparative enrichment scores. (E) Circular inter‐functional module association network. Nodes represent genes; arcs encode pathway category membership (color‐coded). Arc thickness reflects log2 fold‐change. Pathways include: Alzheimer disease, ALS, Chemical carcinogenesis–ROS, COVID‐19, Diabetic cardiomyopathy, Huntington disease, Oxidative phosphorylation, Parkinson disease, Prion disease, and Ribosome. (F) KEGG pathway enrichment dot plot. Top enriched pathways: Ribosome, Coronavirus disease–COVID‐19, Parkinson disease, Oxidative phosphorylation, Huntington disease, Prion disease, Chemical carcinogenesis–ROS, Diabetic cardiomyopathy, ALS, and Alzheimer disease.

GO and KEGG enrichment analyses characterized the transcriptional programs of pediatric AHR airway cells. GO Biological Process analysis (Figure 6A) showed dominant enrichment of cytoplasmic translation, ribosome assembly, ATP metabolic process, ribonucleoprotein complex biogenesis, oxidative phosphorylation, aerobic respiration, ribonucleoprotein complex subunit organization, and ribosome biogenesis—collectively reflecting heightened biosynthetic and metabolic activity in hyperresponsive cells.

GO Cellular Component analysis (Figure 6B) localized enriched genes to cytosolic ribosome, ribosomal subunit, ribosome, focal adhesion, and cell‐substrate junction compartments. GO Molecular Function analysis (Figure 6C) identified structural constituent of ribosome, cadherin binding, rRNA binding, and NADH dehydrogenase activities as the most significant categories. An integrated horizontal bar chart (Figure 6D) compared enrichment scores across all three GO ontology domains.

KEGG pathway analysis (Figure 6F) identified Ribosome, Coronavirus disease‐COVID‐19, Parkinson disease, Oxidative phosphorylation, Huntington disease, Prion disease, Chemical carcinogenesis‐ROS, Diabetic cardiomyopathy, ALS, and Alzheimer disease among the top enriched pathways. Because KEGG neurodegenerative disease terms often share oxidative phosphorylation, mitochondrial, and ribosomal genes, these results were interpreted primarily as evidence of metabolic and oxidative stress‐related transcriptional activity rather than direct involvement of neurodegenerative disease mechanisms in pediatric AHR. The circular inter‐functional module association network (Figure 6E) visualized genes shared across pathway categories, with arc thickness reflecting log2 fold‐change magnitude.

### 4.7. Network pharmacology and target identification of pediatric airway hyperresponsiveness

To extend the single‐cell findings into a therapeutic hypothesis framework, network pharmacology was used to explore candidate herbs, compounds, and targets related to pediatric airway hyperresponsiveness. This analysis was designed as an exploratory bridge between scRNA‐seq‐derived disease signals and compound‐target prediction, rather than as direct evidence of therapeutic efficacy.

**Figure 7:**
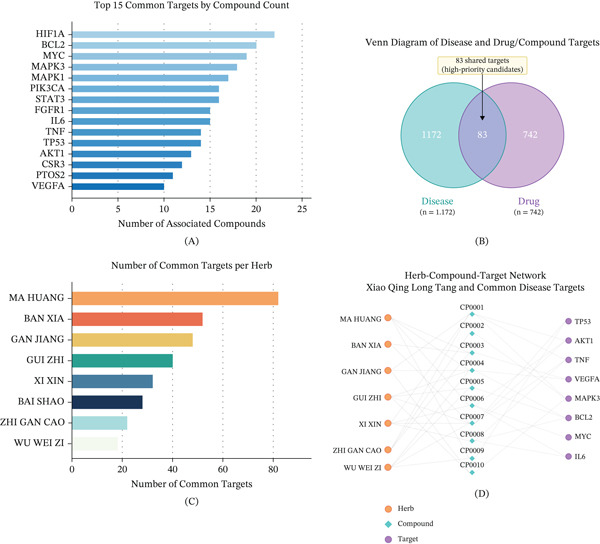
Network pharmacology analysis and therapeutic target identification for pediatric airway hyperresponsiveness. (A) Horizontal bar chart of the top 15 disease‐associated target genes ranked by number of associated compounds (Xiao Qing Long Tang). Top targets: VEGFA, PTGS2, ESR1, AKT1, TP53, TNF, IL6, STAT3, EGFR, PIK3CA, MAPK1, MAPK3, MYC, BCL2, and HIF1A. (B) Venn diagram showing the intersection between disease‐associated targets (1,172) and drug/compound targets (742), yielding 83 shared targets as high‐priority therapeutic candidates. (C) Bar chart of common target counts per herb. (D) Herb–compound–target network. Teal nodes = herb ingredients; yellow nodes = compounds (PubChem CIDs); dark nodes = target genes. Edge width reflects interaction strength. Hub targets include FOS, SOD1, NOS2, CAV1, CDK1, CYCS, HMGB1, VEGFA, and PTGS2.

Using the single‐cell results as a biological starting point, network pharmacology analysis prioritized potential therapeutic targets and molecular interactions in pediatric AHR. Figure 7A lists the top 15 frequently targeted genes, with VEGFA, PTGS2, and ESR1 ranking highly among candidate targets. Figure 7B shows the intersection between disease‐associated targets (1,172) and drug‐associated targets (742), yielding 83 shared candidates. In Figure 7C, MA HUANG (~80 targets) and BAN XIA (~65 targets) showed the broadest target coverage among the herbs examined. The herb‐compound‐target network in Figure 7D illustrates a multi‐component, multi‐target structure; however, these computational links should be viewed as candidates for further docking, perturbation, and in vivo validation.

### 4.8. Herb‐target and compound‐target relationships in the exploratory network pharmacology analysis

**Figure 8:**
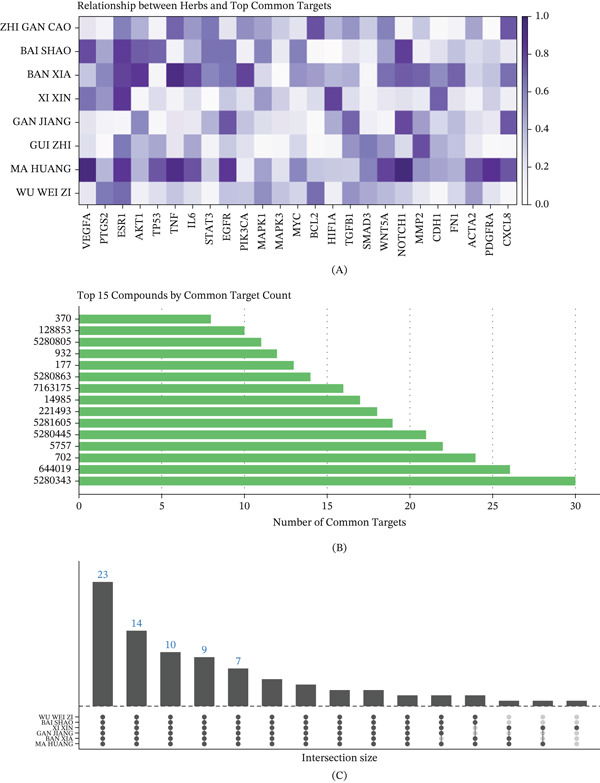
Herb–target interaction and active compound analysis for pediatric airway hyperresponsiveness treatment. (A) Hierarchical clustering heat map of herb–target binding intensity; purple intensity represents interaction strength (0–1); (B) Top 15 compounds ranked by number of common targets; (C) UpSet plot and bar chart showing target overlap among multiple herbs, revealing synergistic mechanisms.

This analysis summarizes predicted associations between herbal constituents and candidate therapeutic targets for pediatric AHR. In Figure 8A, herb‐target interaction strength is displayed as a hierarchical clustering heat map, with darker purple indicating stronger predicted interactions. BAN XIA, GAN JIANG, and MA HUANG showed broader predicted target coverage, whereas WU WEI ZI, ZHI GAN CAO, and GUI ZHI appeared more concentrated around selected gene groups. Figure 8B ranks the top 15 compounds by the number of shared predicted targets, with compound 5280343 showing the highest multi‐target potential (~25 targets), followed by compounds 440819 and 702. Figure 8C uses an UpSet plot to summarize overlap among herbs, with the largest intersection containing 23 shared targets. These patterns suggest possible complementary target coverage within the formula, but they do not establish pharmacological activity without experimental confirmation.

## 5. Discussion

This study provides a single‐cell atlas‐style analysis of pediatric airway hyperresponsiveness mucosa and maps the major epithelial and immune‐associated transcriptional programs present in this public dataset. Rather than claiming a fundamentally new disease‐driving mechanism, the main value of the work lies in organizing known AHR‐relevant inflammatory, epithelial, remodeling, and macrophage‐associated signals into a cell‐resolved framework. This revised interpretation is more consistent with the current evidence base and with the limitations of a single public scRNA‐seq dataset.

The prominent representation of basal cells in our atlas is consistent with their established role as multipotent progenitors of the airway epithelium, capable of self‐renewal and differentiation into specialized epithelial subtypes following injury [10, 11]. Under hyperresponsive conditions, the altered transcriptional state of basal cells observed in our pseudotime analysis suggests disruption of normal epithelial homeostasis, potentially reflecting aberrant activation of repair programs in the chronically inflamed airway. The concurrent presence of ciliated cells and goblet cells at altered proportions is consistent with the mucociliary dysfunction characteristic of AHR, where impaired mucus clearance and pathological mucus production together sustain the inflammatory microenvironment [12].

The differential expression of TSLP and IL33 in our single‐cell atlas provides molecular evidence for alarmin‐driven epithelial‐immune crosstalk in the hyperresponsive airway epithelium. TSLP expression, predominantly enriched in immune cell clusters in our atlas, represents a key orchestrator of type 2 inflammatory responses by promoting dendritic cell activation and Th2 cell differentiation [13, 14]. Its elevated expression in AHR may reflect sustained epithelial stress signaling as part of an aberrant innate immune activation response to chronic inflammatory damage. IL33 has established roles in innate lymphoid cell type 2 (ILC2) activation, mast cell priming, and eosinophilic inflammation [15], while its spatially coordinated expression with TSLP suggests cooperative alarmin signaling that amplifies downstream type 2 immune cascades [16, 17]. The coordinated upregulation of these epithelial alarmins in disease‐specific cellular subpopulations suggests their combined contribution to the cytokine‐mediated airway remodeling characteristic of pediatric AHR.

The expression pattern of CCL26 and FCGR3B in our single‐cell data highlights complementary mechanisms for eosinophilic and neutrophilic inflammation in hyperresponsive airways. CCL26 (eotaxin‐3) functions as a potent eosinophil chemoattractant, orchestrating eosinophilic tissue infiltration through CCR3 receptor engagement on circulating eosinophils [18, 19]. FCGR3B enrichment in the immune cell cluster further implicates neutrophil‐mediated innate responses as a concurrent inflammatory mechanism in pediatric AHR. The cellular sources and transcriptional regulators of these chemotactic signals in hyperresponsive airway epithelium represent promising targets for precision anti‐inflammatory strategies. However, causal relationships between CCL26/FCGR3B expression patterns and granulocytic airway disease phenotypes require validation through functional perturbation models.

The enriched expression of POSTN and CLCA1 in specific cellular subpopulations points toward active subepithelial remodeling and goblet cell‐driven mucus pathology as molecular features of hyperresponsive airways. Periostin (POSTN), a matricellular protein secreted by fibroblasts and epithelial cells, contributes to subepithelial fibrosis, basement membrane thickening, and TGF‐*β*‐mediated tissue remodeling that collectively drive airway wall stiffening in chronic disease [20, 21]. CLCA1, a chloride channel accessory protein expressed specifically in goblet cells, regulates mucus production and MUC5AC secretion, and may modulate airway surface hydration and mucus viscoelasticity relevant to mucociliary dysfunction [22, 23]. The spatially coordinated expression of these secretory and matrix‐modifying molecules across multiple cell types suggests a multicellular remodeling program that extends beyond any single cell population.

The identification of 23 macrophage‐associated subpopulations suggests substantial innate immune heterogeneity within the pediatric AHR airway mucosa. At the same time, this result must be interpreted with caution. High‐resolution clustering can over‐partition continuous transcriptional gradients, particularly when some clusters contain small cell numbers or overlapping marker profiles. Therefore, the M1‐M23 labels are best understood as computationally defined macrophage states. Their consolidation into broader pro‐inflammatory, anti‐inflammatory, and tissue‐remodeling categories provides a practical interpretive framework, but independent dataset replication, trajectory stability testing, and functional validation will be necessary before these states can be considered stable biological subtypes [24, 25].

GO and KEGG enrichment analyses showed prominent cytoplasmic translation, ribosome biogenesis, oxidative phosphorylation, and mitochondrial pathway signatures, indicating increased biosynthetic and metabolic activity in the analyzed airway cells. These findings are consistent with the energetic demands of inflammation, epithelial repair, and mucus production [26]. However, the enrichment of Parkinson disease, Alzheimer disease, and Huntington disease pathways should not be overinterpreted as evidence of neurodegenerative biology in pediatric AHR. These KEGG terms are often driven by broadly expressed oxidative phosphorylation, mitochondrial, and ribosomal genes, and are therefore better framed as indirect indicators of mitochondrial and oxidative‐stress‐related transcriptional activity [27].

Network pharmacology identified VEGFA, PTGS2, and ESR1 as prioritized targets within the overlap between disease‐associated and compound‐associated networks, with additional candidates including AKT1, TP53, TNF, IL6, STAT3, EGFR, and PIK3CA. This analysis is useful for generating therapeutic hypotheses and for connecting transcriptomic signatures with possible compound‐target relationships. Nevertheless, the transition from scRNA‐seq findings to Xiao Qing Long Tang target prediction remains computational and should be interpreted in line with broader multi‐omics and network‐pharmacology principles [28, 29]. In the absence of molecular docking, perturbation assays, animal models, or clinical validation, these targets should be considered candidates for future testing rather than confirmed therapeutic mechanisms [30].

Pseudotime analysis offered a possible transcriptional ordering of epithelial states in hyperresponsive airways. The presence of cell‐type‐specific transcriptional programs suggests that airway hyperreactivity may be associated with altered epithelial homeostasis or repair. However, these signatures are computational inferences from cross‐sectional data and do not prove lineage transitions or causal differentiation pathways. In vitro differentiation models, lineage‐tracing approaches, or longitudinal sampling would be required to confirm whether the inferred trajectories reflect true biological progression. More broadly, computational transcriptomic analyses should be interpreted with awareness of technical and biological sources of bias, particularly when differential expression and pathway enrichment are used to support mechanistic claims [31].

## 6. Limitations

Several limitations should be considered when interpreting these findings. First, the analysis was based on a single public GEO dataset and did not include matched healthy pediatric controls or an independent validation cohort. This limits the ability to distinguish disease‐specific changes from baseline pediatric airway variation, dataset‐specific effects, or technical artifacts. Second, pseudotime trajectories, macrophage polarization states, and pathway‐level interpretations remain computational inferences. Future work should integrate healthy pediatric airway scRNA‐seq data, independent AHR/asthma cohorts, and experimental validation to test the robustness and biological relevance of the reported signatures.

In addition, bronchial brushing samples primarily represent the mucosal surface and may not fully capture deeper airway compartments such as smooth muscle, submucosal glands, or peribronchial stromal tissue. The macrophage subclustering may also be over‐resolved, and some M1‐M23 groups may correspond to transitional, low‐abundance, or partially redundant states. Finally, the network pharmacology section provides target prioritization only; it does not demonstrate binding, pathway modulation, or therapeutic efficacy. Molecular docking, cellular perturbation, animal models, and eventually clinical validation will be required before these predicted herb‐compound‐target relationships can be treated as mechanistic evidence.

## 7. Conclusion

In conclusion, this study presents a cell‐resolved atlas of pediatric AHR mucosa and highlights epithelial inflammatory signals, macrophage‐associated transcriptional diversity, metabolic pathway activity, and candidate compound‐target networks. The findings refine the organization of known AHR‐related programs within a pediatric single‐cell context and provide a basis for future hypothesis‐driven work. Their translational value will depend on validation in healthy controls, independent cohorts, and functional models capable of testing causality.

## Funding

No funding was received for this manuscript.

## Ethics Statement

The authors have nothing to report.

## Conflicts of Interest

The authors declare no conflicts of interest.

## Supporting information


**Supporting Information 1** Additional supporting information can be found online in the Supporting Information section. 

## Data Availability

The data that support the findings of this study are available from the corresponding authors upon reasonable request.
