# Supplementary material for: Single Cell Transcriptomic Atlas Reveals Cellular Heterogeneity and Molecular Mechanisms in Pediatric Airway Hyperresponsiveness Epithelium
Source: Int J Genomics. 2026 Jun 9;2026:3815412. doi: 10.1155/ijog/3815412 (PMC13247550; doi:10.1155/ijog/3815412)
Supplement: Supplementary file 1 — Supporting Information 1 Additional supporting information can be found online in the Supporting Information section. [file IJOG-2026-3815412-s001.docx]

# **Supplementary Materials**

Single Cell Transcriptomic Atlas Reveals Cellular Heterogeneity and Molecular Mechanisms in Pediatric Airway Hyperresponsiveness Epithelium

*Weiming Yang, Shunfeng Mao, Liuyang Zhou**

## Table of Contents

- Table S1: Clinical and demographic characteristics of study cohort
- Table S2: Highly variable genes identified in scRNA-seq analysis
- Table S3: Cell type-specific marker genes
- Table S4: Top enriched GO terms and KEGG pathways
- Table S5: Macrophage subpopulation characteristics
- Supplementary Methods: Detailed experimental protocols

## Table S1: Clinical and demographic characteristics of study cohort

*Summary of patient demographics, clinical features, and sample collection details for the pediatric airway hyperresponsiveness cohort used in single-cell RNA sequencing analysis.*

| **Characteristic** | **Value/Description** |
| --- | --- |
| Sample size (n) | 15 patients |
| Age range | 5-14 years |
| Mean age ± SD | 8.7 ± 2.3 years |
| Sex (Male/Female) | 9/6 (60%/40%) |
| Diagnostic criteria | Methacholine PC20 < 8 mg/mL |
| Mean PC20 value | 4.2 ± 1.8 mg/mL |
| Comorbid allergic rhinitis | 11 (73.3%) |
| Comorbid eczema | 5 (33.3%) |
| Current ICS use | 12 (80%) |
| Current LABA use | 7 (46.7%) |
| Sample collection method | Bronchial brushing |
| Collection site | Main bronchi and lobar bronchi |
| Cell viability | > 85% |
| Total cells sequenced | 47,523 |
| Mean cells per sample | 3,168 ± 892 |
| Sequencing platform | 10x Genomics Chromium |
| CD45 depletion | No (comprehensive epithelial-immune profiling) |

*ICS: inhaled corticosteroids; LABA: long-acting beta-agonists; PC20: provocative concentration causing 20% fall in FEV1; SD: standard deviation.*

## Table S2: Highly variable genes identified in scRNA-seq analysis

*Top 50 highly variable genes showing elevated expression and high variability across cell populations. These genes were selected based on mean expression levels and normalized variance.*

| **Gene Symbol** | **Mean Expression** | **Variance** | **Functional Category** |
| --- | --- | --- | --- |
| KRT16 | 4.82 | 12.35 | Epithelial differentiation |
| KRT17 | 4.65 | 11.89 | Epithelial differentiation |
| KRT14 | 4.43 | 10.92 | Basal cell marker |
| SPRR3 | 3.98 | 9.87 | Inflammatory response |
| MSMB | 3.76 | 9.45 | Inflammatory signaling |
| CXCL5 | 3.54 | 8.93 | Chemokine signaling |
| MMP10 | 3.42 | 8.67 | Tissue remodeling |
| ADAM12 | 3.31 | 8.45 | Extracellular matrix |
| MALAT1 | 3.28 | 8.32 | Long non-coding RNA |
| S100A8 | 3.15 | 7.98 | Calcium binding |
| S100A9 | 3.09 | 7.76 | Immune response |
| CXCL8 | 2.98 | 7.54 | Neutrophil chemotaxis |
| IL1B | 2.87 | 7.32 | Pro-inflammatory cytokine |
| TNF | 2.76 | 7.11 | Inflammatory mediator |
| CCL20 | 2.68 | 6.89 | Chemokine |
| MUC5AC | 2.54 | 6.67 | Mucus production |
| MUC5B | 2.43 | 6.45 | Mucus secretion |
| FOXJ1 | 2.35 | 6.28 | Ciliated cell marker |
| SCGB1A1 | 2.28 | 6.12 | Club cell marker |
| CFTR | 2.21 | 5.98 | Ion channel |

*Expression values are log2-transformed normalized counts. Variance is calculated after normalization and represents gene-specific variability across all cells.*

## Table S3: Cell type-specific marker genes

*Canonical marker genes used for identification and validation of major cell types in the airway mucosa single-cell atlas.*

| **Cell Type** | **Marker Genes** | **Biological Function** |
| --- | --- | --- |
| Basal cells | KRT5, KRT14, TP63, NGFR | Progenitor/stem cells, epithelial renewal |
| Ciliated cells | FOXJ1, DNAH5, TUBA1A, RSPH1 | Mucociliary clearance, airway defense |
| Goblet cells | MUC5AC, MUC5B, TFF3, SPDEF | Mucus production and secretion |
| Club cells | SCGB1A1, SCGB3A1, CYP2F1, LYPD2 | Secretory function, xenobiotic metabolism |
| Ionocytes | FOXI1, CFTR, ATP6V1B1, ASCL3 | Ion transport, airway surface liquid regulation |
| Deuterosomal cells | DEUP1, CCNO, CDC20B, PLK4 | Centriole amplification, ciliogenesis |
| Eosinophils | SIGLEC8, IL5RA, CCR3, GATA1 | Type 2 inflammation, allergic response |
| Macrophages (M1) | CD68, MARCO, NOS2, TNF | Pro-inflammatory response |
| Macrophages (M2) | CD163, MRC1, ARG1, IL10 | Anti-inflammatory, tissue repair |
| T cells | CD3E, CD3D, CD4, CD8A | Adaptive immunity |
| B cells | CD19, MS4A1, CD79A, JCHAIN | Antibody production |

## Table S4: Top enriched GO terms and KEGG pathways

*Gene Ontology biological processes and KEGG pathways significantly enriched in airway hyperresponsiveness. FDR: false discovery rate.*

| **Category** | **Term/Pathway** | **Gene Count** | **FDR** |
| --- | --- | --- | --- |
| GO:BP | Inflammatory response | 156 | 1.2e-45 |
| GO:BP | Immune system process | 234 | 3.4e-42 |
| GO:BP | Regulation of smooth muscle contraction | 87 | 2.1e-38 |
| GO:BP | Extracellular matrix organization | 92 | 5.6e-35 |
| GO:BP | Cell adhesion | 145 | 7.8e-33 |
| GO:BP | Cytokine-mediated signaling | 118 | 1.3e-31 |
| GO:BP | Leukocyte migration | 76 | 4.5e-29 |
| GO:BP | Wound healing | 89 | 6.7e-27 |
| KEGG | IL-17 signaling pathway | 45 | 8.9e-26 |
| KEGG | TNF signaling pathway | 52 | 1.1e-24 |
| KEGG | NF-kappa B signaling pathway | 48 | 2.3e-23 |
| KEGG | Chemokine signaling pathway | 67 | 4.5e-22 |
| KEGG | Cytokine-cytokine receptor interaction | 89 | 5.6e-21 |
| KEGG | ECM-receptor interaction | 43 | 7.8e-20 |
| KEGG | Focal adhesion | 56 | 9.2e-19 |
| KEGG | Calcium signaling pathway | 61 | 1.4e-18 |
| KEGG | MAPK signaling pathway | 72 | 2.8e-17 |
| KEGG | JAK-STAT signaling pathway | 54 | 3.9e-16 |

## Table S5: Macrophage subpopulation characteristics

*Summary of 23 macrophage subpopulations (M1-M23) identified through high-resolution clustering, with their functional classification and representative markers.*

| **Subpopulation** | **Cell Count** | **Functional Category** | **Key Markers** |
| --- | --- | --- | --- |
| M1 | 892 | Pro-inflammatory | TNF, IL1B, CXCL8, NOS2 |
| M2 | 756 | Pro-inflammatory | CCL3, CCL4, IL6, CXCL10 |
| M3 | 634 | Pro-inflammatory | IL23A, IL12B, CD86 |
| M4 | 521 | Anti-inflammatory | IL10, CD163, MRC1, MSR1 |
| M5 | 487 | Anti-inflammatory | ARG1, CCL18, CD209, STAB1 |
| M6 | 445 | Tissue remodeling | MMP9, MMP12, TIMP1 |
| M7 | 412 | Tissue remodeling | SPP1, TREM2, APOE |
| M8 | 389 | Pro-inflammatory | CXCL9, CXCL11, IDO1 |
| M9 | 356 | Antigen presentation | HLA-DRA, HLA-DRB1, CD74 |
| M10 | 334 | Phagocytic | MARCO, MSR1, CD36 |
| M11-M23 | 2,187 | Mixed phenotypes | Various markers |

*Subpopulations M11-M23 represent transitional or mixed phenotype clusters with lower cell counts (ranging from 98 to 245 cells each). Complete marker profiles for all 23 subpopulations are available upon request.*

## Supplementary Methods

### Quality Control Parameters

Cells were filtered based on the following criteria: (1) number of detected genes between 200 and 6,000; (2) total UMI counts between 500 and 50,000; (3) mitochondrial gene percentage < 15%; (4) doublet score < 0.25 using Scrublet algorithm. Genes expressed in fewer than 3 cells were excluded from downstream analysis.

### Normalization and Batch Correction

Raw count data were normalized using the NormalizeData function in Seurat v4.3.0 with a scale factor of 10,000. Log-transformation was applied to the normalized data. Batch effects were corrected using Harmony algorithm with default parameters, integrating samples based on patient ID as the batch variable.

### Clustering and Cell Type Annotation

Graph-based clustering was performed using the Louvain algorithm implemented in Seurat FindClusters function. Resolution parameter was set to 0.8 for main cell types and 1.2 for macrophage subclustering. Cell type identities were assigned based on expression of canonical marker genes, with additional validation using SingleR against reference datasets (Human Primary Cell Atlas and Blueprint/ENCODE).

### Differential Expression Analysis

Differential gene expression between cell types was performed using Wilcoxon rank-sum test in Seurat FindMarkers function. Genes with adjusted p-value (Bonferroni correction) < 0.05 and log2 fold change > 0.5 were considered significant. For each cell type, top marker genes were selected based on combined ranking of fold change and statistical significance.

### Trajectory Analysis

Pseudotime trajectory analysis was conducted using Monocle 3. Basal cells were selected as the root of the trajectory based on their progenitor characteristics. Cells were ordered along pseudotime using reversed graph embedding, and genes significantly associated with pseudotime progression were identified using graph-test with q-value < 0.05.

### Functional Enrichment Analysis

Gene Ontology (GO) enrichment analysis was performed using clusterProfiler package v4.6.2. Biological process terms were tested for over-representation using hypergeometric test with Benjamini-Hochberg FDR correction. KEGG pathway analysis was conducted using enrichKEGG function with organism set to 'hsa' (Homo sapiens). Only terms/pathways with FDR < 0.05 and gene count > 5 were considered significant.

### Statistical Analysis

All statistical analyses were performed in R version 4.2.1. Two-tailed tests were used throughout unless otherwise specified. Multiple testing correction was applied using Benjamini-Hochberg method. Statistical significance threshold was set at FDR < 0.05. Data visualization was performed using ggplot2, Seurat visualization functions, and custom R scripts.

### Software and Code Availability

Data processing: Cell Ranger v7.0.0, Seurat v4.3.0; Batch correction: Harmony v0.1.1; Trajectory analysis: Monocle 3 v1.3.1; Functional analysis: clusterProfiler v4.6.2, enrichplot v1.18.3; Visualization: ggplot2 v3.4.0, ComplexHeatmap v2.14.0, pheatmap v1.0.12. Custom analysis scripts are available upon reasonable request.
